# Supplementary material for: The causal role of circulating inflammatory markers in osteoporosis: a bidirectional Mendelian randomized study
Source: Front Immunol. 2024 Jul 18;15:1412298. doi: 10.3389/fimmu.2024.1412298 (PMC11291241; doi:10.3389/fimmu.2024.1412298)
Supplement: Supplementary file 1 [file DataSheet_1.zip › supplementary material/CIM-OSTEOPOROSIS /GCST90274822.csv_leave_one_out.pdf]

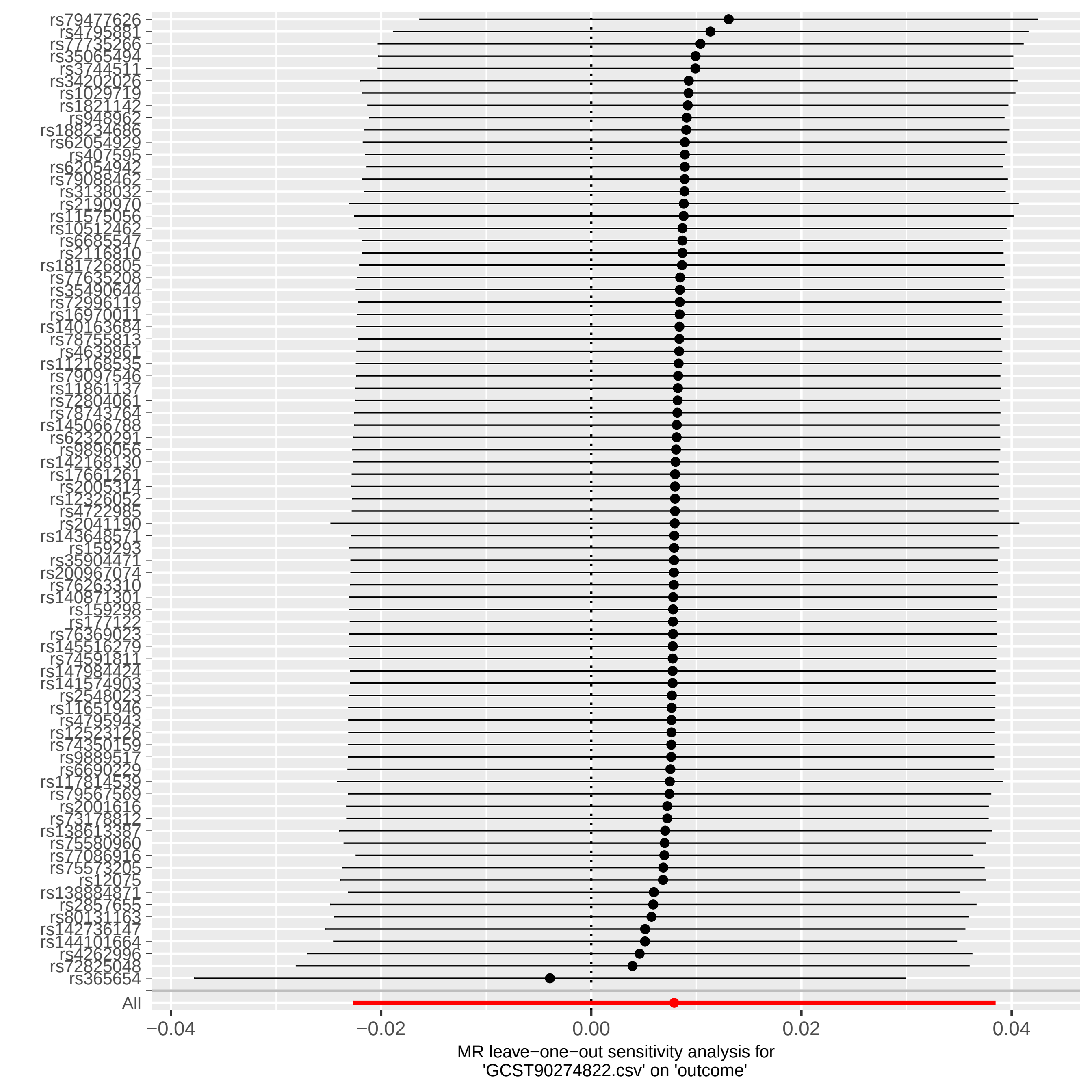
rs79477626
rs4795881
rs77735266
rs35065494
rs3744511
rs34202026
rs1029719
rs1821142
rs948962
rs188234686
rs62054929
rs407595
rs62054942
rs79088462
rs3138032
rs2190970
rs11575056
rs10512462
rs6685547
rs2116810
rs181726805
rs77635208
rs35490644
rs72996119
rs16970011
rs140163684
rs78755813
rs4639861
rs112168535
rs79097546
rs11861137
rs72804061
rs78743764
rs145066788
rs62320291
rs9896056
rs142168130
rs17661261
rs2005314
rs12326052
rs4722985
rs2041190
rs143648571
rs159293
rs35904471
rs200967074
rs76263310
rs140871301
rs159298
rs177122
rs76369023
rs145516279
rs74591811
rs147984424
rs141574903
rs2548023
rs11651946
rs4795943
rs12523126
rs74350159
rs9889517
rs6690229
rs117814539
rs79567569
rs2001616
rs73178812
rs138613387
rs75580960
rs77086916
rs75573205
rs12075
rs138884871
rs2857655
rs80131163
rs142736147
rs144101664
rs4262996
rs72825048
rs365654
All
−0.04
−0.02
0.00
0.02
0.04
MR leave−one−out sensitivity analysis for
'GCST90274822.csv' on 'outcome'
